# Navigating Life With High‐Grade Glioma: Experiences and Needs of Adolescents and Young Adults

**DOI:** 10.1002/cam4.70867

**Published:** 2025-04-08

**Authors:** Kaviya Devaraja, Maureen Daniels, Derek S. Tsang, Kim Edelstein, Julie Bennett, Cheryl Kanter, Warren Mason, Abha A. Gupta, Jonathan Avery

**Affiliations:** ^1^ Institute of Medical Science University of Toronto Toronto Canada; ^2^ Department of Supportive Care, Adolescent and Young Adult Program, Princess Margaret Cancer Centre University of Toronto Toronto Canada; ^3^ Department of Psychiatry University of Toronto Toronto Canada; ^4^ Radiation Medicine Program, Princess Margaret Cancer Centre University Health Network Toronto Canada; ^5^ Department of Supportive Care, Princess Margaret Cancer Centre University Health Network Toronto Canada; ^6^ Division of Medical Oncology and Hematology, Princess Margaret Cancer Centre University of Toronto Toronto Canada; ^7^ Division of Hematology and Oncology The Hospital for Sick Children Toronto Canada; ^8^ Divisions of Neurology and Medical Oncology, Department of Medicine, Princess Margaret Cancer Centre University of Toronto Toronto Canada; ^9^ School of Nursing University of British Columbia Vancouver Canada

**Keywords:** adolescent and young adult, brain tumor, cancer, cognitive impairments, high‐grade glioma, mixed‐methods study, oncology, psychosocial challenges, support programs

## Abstract

**Background:**

Adolescents and young adults (AYA, 18–39) with high‐grade glioma (HGG) face unique challenges at a life stage focused on autonomy, careers, relationships, and family planning.

**Aim:**

This study explores their experiences to inform life‐stage appropriate support and resources.

**Methods:**

In this mixed‐methods study, we surveyed AYA HGG patients at Princess Margaret Cancer Centre (PM) to assess symptom experiences and care satisfaction. Interviews further explored their illness experiences and needs. Descriptive statistics summarized survey data, and thematic analysis guided by Braun and Clarke's framework identified key interview themes. Triangulation compared survey and interview results for a comprehensive understanding.

**Results:**

Seventeen participants (7 men, 10 women; mean age 30.57) completed surveys and interviews. Triangulation revealed typical AYA challenges, such as delays in education, careers, and relationships, along with HGG‐specific issues. Three main themes emerged: (1) managing cognitive and treatment‐related impacts on life goals, (2) addressing physical and cognitive impairments affecting relationships, and (3) navigating identity loss and independence due to neurological symptoms.

**Conclusions:**

These findings highlight the need for tailored interventions and educational support integrated into AYA HGG care pathways.

## Abstract 2

Adolescents and young adults (AYAs) with high‐grade glioma (HGG) face unique challenges at critical life stages, including disruptions to education, careers, relationships, and independence due to cognitive and physical impairments. This mixed‐methods study, combining surveys and interviews, identified three key themes: managing the impact of symptoms on life goals, addressing social and relational challenges, and coping with identity loss and dependency. Findings highlight the need for tailored interventions and support programs to address the specific needs of AYAs with HGG.

## 1 Background

High‐grade gliomas (HGGs) are aggressive brain tumors that are generally incurable [1]. In Canada, an estimated 3000 new cases of HGG are diagnosed annually, representing a substantial burden among brain tumors [2]. The most common type of HGG is glioblastoma (GBM), comprising approximately 12%–15% of all intracranial tumors and 50%–60% of all astrocytic tumors diagnosed in Canada [3]. Despite treatment advancements, the prognosis for HGG remains poor, with median survival around 12–15 months, peaking in adults aged 45–75 years [4].

HGG are relatively rare in adolescent young adults (AYAs), comprising 5.9% of primary brain tumors in this population [5]. AYAs, aged 15–39 years, are at critical life stages involving education, career progression, identity development, and personal relationships; individuals in this age group face unique psychosocial and developmental challenges [6]. AYAs generally have better long‐term survival compared to older adults, with median survival up to several years [7, 8]. In Canada, approximately 8000 AYAs are diagnosed with cancer annually, with HGG being a rare but significant subtype due to their poor prognosis [9]. Although AYAs with HGG experience similar symptoms as older adults with HGG, such as headaches, seizures, cognitive changes, fatigue, and nausea, the psychosocial impact on AYAs differs [10]. The sudden diagnosis of cancer disrupts their developmental milestones, imposing physical, emotional, and social burdens that are different when compared to older adults who are at a more mature, stable stage of life [11]. Increasingly, it is being recognized that many of these younger patients have a cancer predisposition syndrome, adding to their anxiety and stress about family planning, future health risks, and the potential impact on their relatives [12].

AYA cancer care is divided between pediatric and adult oncology services. In Canada, pediatric hospitals provide treatment, follow‐up, and psychosocial support until age 18, at which point patients transition to adult oncology services. Adult hospitals provide oncologic care with disease‐focused teams but are burdened with high patient volumes and staffing challenges, which may limit the ability to fully address the specific care needs of AYAs with cancer, creating a gap in support between pediatric and adult centers [13]. This deficit extends to support programs, facilities, and advocacy initiatives targeting AYA HGG patients [14], resulting in an absence of life‐stage specific care for AYAs [15].

The PM AYA program was established in 2014 to optimize and standardize supportive care delivery that is tailored to the needs of AYA patients. The aim of this study was to explore the illness experiences and challenges of AYA HGG patients, to inform the development of resources to address their specific needs and mitigate existing gaps in care through the PM AYA program.

## 2 Methods

In this cross‐sectional study, a mixed‐methods simultaneous quantitative‐qualitative approach was used, including an online self‐reported participant survey and virtual one‐on‐one interviews, to explore experiences and challenges navigating the cancer care system of AYA HGG patients. Ethics approval was obtained from the University Health Network (UHN) research ethics board (CAPCR #22‐6012).

### 2.1 Study Participants and Setting

Patients were eligible if they spoke English, were aged 18–39 years at the time of enrolment, and had a histologic diagnosis of HGG. This age range was selected as it captures the unique developmental and treatment‐related challenges faced by AYAs with HGG [16]. Eligible HGG diagnoses included: GBM isocitrate dehydrogenase (IDH)‐wildtype, astrocytoma IDH‐mutated World Health Organization (WHO) grade 4, astrocytoma IDH‐mutated WHO grade 3, anaplastic oligodendroglioma WHO grade 3, or diffuse midline glioma WHO grade 4. The study included AYAs at different stages of treatment to capture a range of experiences. Patients were enrolled from the neuro‐oncology clinic at a tertiary care center (Princess Margaret Cancer Centre, University Health Network).

### 2.2 Participant Recruitment

**FIGURE 1:**
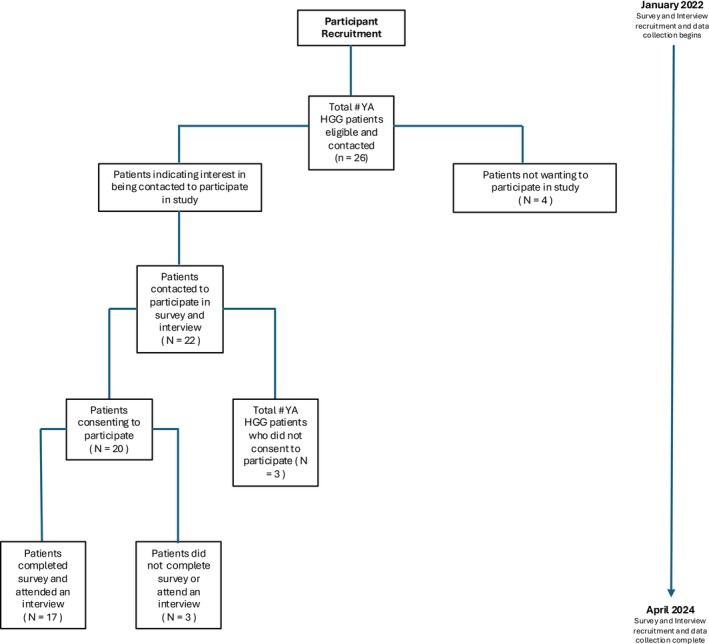
Participant recruitment flow chart.

AYAs were informed of the study by clinical teams at the PM, including oncologists, registered nurses, neuropsychologists, and social workers from the neuro‐oncology clinic and AYA clinic. Interested participants were contacted to confirm eligibility, obtain informed consent, and schedule an interview. Participants then received an online survey, which was completed before a virtual interview took place via Microsoft Teams (MS Teams). A sample of 15–20 AYAs was sought to achieve theme saturation [17]. Data collection and recruitment occurred between January 2022 and March 2024 (Figure 1).

### 2.3 Data Collection

#### 2.3.1 Surveys

The survey had two sections: (1) demographics and (2) AYA care (Table S1). Demographic questions assessed sample heterogeneity. The AYA care section explored informational, physical, psychosocial, resource, advocacy, and awareness concerns, care adequacy, and ideas for future solutions. The questions in the AYA care section were developed based on a comprehensive review of the literature [18, 19] and feedback from members of the study team to ensure that the survey accurately captured the multifaceted aspects of AYA care needs. Survey responses were reported using descriptive statistics for continuous variables and frequencies for categorical variables.

#### 2.3.2 Interviews

One‐on‐one interviews, conducted by K.D. via Microsoft Teams, followed a structured interview guide informed by survey questions (Table S2).

Interviews were audio recorded, transcribed verbatim, and identifiers were removed [20]. The questions in the interview guide were developed based on a review of existing literature [21] and feedback from the study team of oncologists and researchers to ensure relevance and comprehensiveness. Thereafter, interviews were analyzed using thematic analysis informed by Braun and Clarke to identify patterns within the transcripts [22]. The analytic process involved familiarization with the data, generating initial codes, and organizing codes into themes and categories using NVivo 10 software. Survey data were then compared with interview findings for a comprehensive understanding of the patients' experiences.

## 3 Results

Seventeen AYAs completed the survey, each also participating in a one‐on‐one interview. Participants ranged in age from 19 to 39 years, with most between 30 and 39 years (*n* = 13, 76.4%). The most common tumor types were GBM IDH‐wildtype (*n* = 5, 29.4%) and astrocytoma IDH‐mutated WHO grade 4 (*n* = 6, 35.3%) (Table 1). Triangulation is the process of using multiple data sources or methods to enhance the credibility and validity of research findings. The triangulation of survey data and interview content revealed several common elements across patients. Patients reported typical challenges that AYAs experience when diagnosed with cancer, such as delays in achieving life‐stage appropriate milestones like completing education, advancing in careers, pursuing platonic and romantic partnerships, and having children. However, AYAs with HGG faced additional challenges associated with the cognitive and physical impairments specific to an HGG diagnosis. Three themes emerged that described these disease‐specific challenges: (1) managing cognitive changes, fatigue, and aggressive treatment impacts to prevent further disruptions in career progression and other life goals, (2) addressing the impact of physical and cognitive impairments on social life and relationships, and (3) navigating loss of identity, independence, and feelings of inadequacy due to seizures and mood changes.

**TABLE 1:** Demographics of study participants.

Characteristics	Total patients survey (*n*, %) *N* = 17
*Age*	
18–24	2 (11.8)
25–29	2 (11.8)
30–39	13 (76.5)
*Gender*	
Male	7 (41.2)
Female	10 (58.8)
*Racial/Ethnic Background*	
White	11 (64.7)
South Asian	4 (23.5)
East Asian	2 (11.8)
*Diagnosis*	
Glioblastoma IDH‐wildtype	5 (29.4)
Astrocytoma IDH‐mutated WHO grade 4	6 (35.3)
Astrocytoma IDH‐mutated WHO grade 3	1 (5.9)
Anaplastic oligoastrocytoma WHO grade 3	2 (11.8)
Anaplastic oligodendroglioma WHO grade 3	2 (11.8)
Diffuse midline glioma WHO grade 4	1 (5.9)
*Education Completed*	
High school diploma	3 (17.7)
University or college degree	6 (35.3)
Graduate or professional degree	8 (47.1)
*Time since Diagnosis*	
Less than a year ago	5 (29.4)
1–3 years ago	6 (35.3)
4–6 years ago	4 (23.5)
More than 6 years ago	2 (11.8)
*Cancer Treatment Stage*	
Currently in active treatment	2 (11.8)
Completed treatment and in long‐term follow‐up care	15 (88.2)
*Other Health Conditions*	
None	13 (76.5)
Another cancer diagnosis	1 (5.9)
Mental health (e.g., anxiety)	3 (17.7)
STI/STD	1 (5.9)
Ulcerative Colitis	1 (5.9)
*Employment Status*	
Student	2 (11.7)
Part‐Time	1 (5.9)
Full‐Time	3 (17.7)
On disability	8 (47.1)
I prefer not to answer	3 (17.7)
*Net Household Income*	
Less than $70,000	6 (35.3)
$90,000–$150,000	3 (17.7)
Greater than $150,000	3 (17.7)
I prefer not to answer	5 (29.4)

### 3.1 Managing Cognitive Changes, Fatigue and Aggressive Treatment Impacts to Prevent Further Disruptions in Career Progression and Other Life Goals

Among 17 survey respondents, 82% (*n* = 14) reported persistent fatigue, 76% (*n* = 13) reported cognitive changes, and 76% (*n* = 13) AYAs found living with HGG to be extremely challenging (Table 2). While satisfaction with support during diagnosis (65%, *n* = 11) and treatment (82%, *n* = 14) was high, post‐treatment support showed notable dissatisfaction (41%, *n* = 7). When triangulated with experiences described in the interviews, participants confirmed these impairments hindered their ability to complete education, advance careers, and pursue other life goals and lacked support to navigate these challenges after treatment.

**TABLE 2:** Survey responses.

Question	Selection (*N* %)					
What challenges or concerns do you experience as a high‐grade glioma patient?	Not at all	Rarely	Sometimes/Occasionally	Frequent/Often	Usually/Almost Always	Always
Headaches	5 (29.4)	4 (23.5)	5 (29.4)	3 (17.7)	0 (0.0)	0 (0.0)
Seizures	7 (41.2)	6 (35.3)	4 (23.5)	0 (0.0)	0 (0.0)	0 (0.0)
Cognitive changes (memory, concentration, confusion)	3 (17.7)	1 (5.9)	8 (47.1)	4 (23.5)	1 (5.9)	0 (0.0)
Problems with speech	7 (41.2)	5 (29.4)	2 (11.8)	3 (17.7)	0 (0.0)	0 (0.0)
Changes in mood, behavior and personality	2 (11.8)	5 (29.4)	6 (35.3)	2 (11.8)	0 (0.0)	2 (11.8)
Muscle weakness	4 (23.5)	6 (35.3)	6 (35.3)	1 (5.9)	0 (0.0)	0 (0.0)
Visual deficits (changes in vision such as double or blurred vision)	5 (29.4)	3 (17.7)	5 (29.4)	1 (5.9)	1 (5.9)	2 (11.8)
Loss of balance and difficulty walking	6 (35.3)	6 (35.3)	3 (17.7)	1 (5.9)	1 (5.9)	0 (0.0)
Fertility/Fertility Preservation	6 (35.3)	2 (11.76)	5 (29.4)	1 (5.9)	1 (5.9)	2 (11.8)
Fatigue	1 (5.9)	2 (11.8)	3 (17.7)	6 (35.3)	4 (23.5)	1 (5.89)
Loss of appetite	4 (23.5)	7 (41.2)	3 (17.7)	2 (11.8)	1 (5.9)	0 (0.0)
Nausea and vomiting	8 (47.1)	3 (17.7)	3 (17.7)	2 (11.8)	1 (5.9)	0 (0.0)
Body image	3 (17.7)	5 (29.4)	2 (11.8)	4 (23.5)	1 (5.9)	2 (11.8)
Sexuality/intimacy	6 (35.3)	1 (5.9)	6 (35.3)	2 (11.8)	1 (5.9)	1 (5.9)
Urinary incontinence	9 (52.9)	5 (29.4)	2 (11.8)	0 (0.0)	0 (0.0)	1 (5.9)

	Just enough	Not enough
2 The education about my diagnosis I received from my health care team, was it:	5 (29.4)	12 (70.6)
3 The information I received about symptoms of being diagnosed for high‐grade glioma from my healthcare team, was it:	5 (29.4)	12 (70.6)
4 The education I received from my healthcare team about available treatment options for high‐grade‐glioma, was it:	6 (35.3)	11 (64.7)
5 The education I received from my healthcare team on the resources and support available to me, was it:	8 (47.1)	9 (52.9)

	Strong agree	Agree	Disagree	Neither agree nor disagree
6 Living with high‐grade glioma as a young adult is extremely challenging	11 (64.7)	2 (11.8)	2 (11.8)	2 (11.8)
7 Are you satisfied with the support you received at the cancer center during:	Yes	No	I am not sure	I prefer not to answer
Diagnosis	11 (64.7)	2 (11.8)	0 (0.0)	4 (23.5)
Treatment	14 (82.4)	1 (5.9)	1 (5.9)	1 (5.9)
Post treatment	3 (17.7)	7 (41.2)	4 (23.5)	3 (17.7)
8 We would like to understand what type of support you have accessed and/or are interested in accessing	I've used and attended the program	I've heard of the program and interested (but have yet to attend)	I've heard of the program and am not interested in attending	I haven't heard of the program
Patient information Line (info Line)	1 (5.9)	2 (11.8)	3 (17.7)	11 (64.7)
AYA Meet‐ups	3 (17.7)	4 (23.5)	3 (17.7)	7 (41.2)
AYA Newsletter	5 (29.4)	2 (11.8)	2 (11.8)	8 (47.1)
AYA Social Media (Twitter, Instagram, and Facebook)	3 (17.7)	2 (11.8)	4 (23.5)	8 (47.1)
Young Adult Cancer Canada programs [on‐line community, retreats]	2 (11.8)	4 (23.5)	4 (23.5)	7 (41.2)
Cancer Center Specific Social Media	4 (23.5)	3 (17.7)	5 (29.4)	5 (29.4)
Cancer Center Patient & Family Library	5 (29.4)	5 (29.4)	4 (23.5)	3 (17.7)
Canadian Cancer Society Community Services	5 (29.4)	5 (29.4)	2 (11.8)	5 (29.4)
Cancer Center Patient Education Materials	7 (41.2)	5 (29.4)	2 (11.8)	3 (17.7)
Psychosocial Oncology Service/Program at Cancer Center	4 (23.5)	4 (23.5)	2 (11.8)	7 (41.2)
Wellspring	1 (5.9)	7 (41.2)	4 (23.5)	5 (29.4)
Gilda's Club	3 (17.7)	3 (17.7)	2 (11.8)	9 (52.9)
Brain Tumor Foundation of Canada	7 (41.2)	1 (5.9)	2 (11.8)	7 (41.2)

For example, P001, a 19‐year‐old living with GBM for 6 months reflected, “Due to memory loss from the tumor, I had to take time away from school…” further elaborating that “I would have liked to have someone who could communicate with the school directly and find me a flexible and less intense schedule…. rather than making me stop completely because of my glioma.” This participant reported that their memory loss forced them to take time away from school and would have benefited from support to continue this pursuit rather than delaying their education. P005, a 28‐year‐old living with grade IV astrocytoma for 8 months, expressed similar disruptions and need for supports. P005 stated, “I had to defer [my degree] for a year…. I didn't have the capacity to understand…. or focus…. or the energy to sit through my classes” and needed “academic support and accommodations from my professors…. to manage my coursework alongside my health challenges” because of sequelae associated with an HGG.

Other participants noted that the symptoms from HGG further disrupted the achievement of life‐stage appropriate milestones, from career progression to having children. P003, a 33‐year‐old living with grade IV astrocytoma noted “[I] needed guidance to transition back to work post‐treatment…. because of the mental confusion…,” a common symptom from HGG, and expressed that a “cognitive rehabilitation program that provide strategies and support to work through [mental confusion] would be super helpful….”. P007 a 33‐year‐old living with anaplastic oligoastrocytoma grade III for 4 years, echoed similar experiences, stating, “I wasn't thinking of having kid….if I wanted to have kids in a few years what if I recur down the line….can I still be there and do everything for my kid…. if I have cognitive decline…. constantly tired……. will I need someone to help me and how much help?”. They emphasized wanting someone to “learn my needs…” and to help with “…have these long‐term planning discussions…. when I am older and potentially wanting to have a family, I can see if it is a realistic option for me.” These participants highlight how the specific symptomology of HGG impacted their professional pursuits and other life‐stage‐specific goals, as well as the need for specific and tailored support to manage these challenges, particularly in the post‐treatment phase.

### 3.2 Addressing the Impact of Physical and Cognitive Impairments on Social Life and Relationships

In addition to those survey respondents who experienced cognitive changes (76%; *n* = 13), 71% (*n* = 12) also experienced visual deficits, (76%) (*n* = 13) and muscle weakness, while and 65% (*n* = 11) experienced challenges with sexuality and intimacy (Table 2). Interviews confirmed these impairments lead to social isolation, loss of hobbies, and relationship struggles, all connected to HGG specific symptomology. For example, P013, a 21‐year‐old living with GBM for 11 months expressed, “because of my brain tumour I have vision loss…. I am not able to drive…. which has been incredibly isolating because my friends don't live that close to me…. if I want to go hang out with them, I would have to drive down to them which I can't really do.” They articulated a desire for “vision therapy …. So, I can drive by myself to visit them [friends] when I feel like seeing them and actually having a social life.” Other participants such as P011, 37‐year‐old living with diffuse midline glioma grade IV for 2 years, also expressed social isolation and need for resources specific to their HGG symptomology, saying “I used to love going out with my friends, but ever since the glioma, I'm tired a lot…” emphasizing a need for “…a program to arrange social events…. [where] brain tumor patients that have the same issues and feel disconnected have the option to join.”

For these participants, they desired to connect with other AYAs with an HGG diagnosis. P017, a 35‐year‐old living with GBM for 7 months, explains that “I used to bike everyday…. [but now] I can no longer ride a bike….the cancer affected the balance and coordination in my legs…. adding that exercise programs like specialized biking equipment or balance training for people like me…. that slowly improve strength and balance would be useful…” yet they were not aware of any program. P002, a 38‐year‐old living with anaplastic oligodendroglioma grade III for 1 year also requested tailored supports for HGG AYAs by asking “if the hospital could provide activity packages or suggestions that are safe for me to do from home…. through zoom…. with other people that have similar abilities and challenges with functioning… it would help me make the most out of each day rather than sitting on the couch watching tv for most of the day….” There was an emphasis on connecting with other AYA with a diagnosis of HGG. P015, a 39‐year‐old living with anaplastic oligodendroglioma grade III, initially diagnosed 12 years ago, struggled with parental demands stating and stated, “I want to meet these [other young parents with HGG]…. it would be useful to ask them how they are managing…. no one else understands it more than someone else going through something similar as you” for support and guidance. This participant emphasizes how connecting with other parents facing similar challenges can provide invaluable support and understanding, helping to navigate the complexities of parenting while managing cognitive impairments from HGG.

Thus, based on the survey results and triangulation with interviews, the specific symptoms of HGG made it difficult for our participants to maintain a normal social life and social relationships and suggested that meeting other AYAs with a similar diagnosis would be a way to support them through any social isolation they experience.

### 3.3 Navigating Loss of Identity, Independence, and Feelings of Inadequacy due to Seizures and Mood Changes

Among survey respondents, 59% (*n* = 10) had seizures, and 88% (*n* = 15) faced changes in mood, behavior, and personality (Table 2). Interviews expanded these findings, highlighting that these changes caused a loss of identity independence and a growing sense of inadequacy. This was true for P005, a 28‐year‐old synchronized swimmer, who had to “…hide that part of my identity [being a synchronized swimmer]… because they [her clinicians] are scared that I could have a seizure and die in the pool.” P005 struggled with this sense of loss. Loss of self was also tied to the loss of independence. P008, a 33‐year‐old living with grade IV astrocytoma for 4 years, lost their sense of independence that was brought on my their HGG diagnosis and treatment, explaining that “forgetfulness makes me rely on others…. I get frustrated and demotivated having to depend on others for simple tasks… it makes me feel like a burden.” This loss of independence was tied to feelings of inadequacy, seen here with P009, a 31‐year‐old living with grade IV astrocytoma for 4 years stated. “I have lost my peripheral vision [from my tumor]… and [and now] I miss numbers [at the grocery store], I will think a chocolate is being sold for $2 but at the billing its $12.97…. I feel sort of useless.”

These participants, as was true for others, spoke of needing tailored practical support and more information on HGG survival rates and risk factors to navigate these losses and feelings of inadequacy. P014, a 33‐year‐old with grade III astrocytoma for 9 years desired “age‐specific statistics like survival rates…. treatment outcomes for young people on clinical trials” to “provide clearer expectations and help us make more informed decisions about our future.” Without this type of information, P006 felt “uncertain if I can maintain my current lifestyle…. what if I need around the clock care in the next few months because I have cognitive decline or can no longer take care of myself,” further elaborating that “having easily accessible resources, like an online portal with links to all AYA HGG information…. would make a significant difference….” These feelings were confirmed via our survey results. Survey results revealed gaps in education about glioma diagnosis (71%, *n* = 12), HGG symptoms (71%, *n* = 12), and available resources (53%, *n* = 9) to help manage daily life suggesting that tailor resources about the impact of HGG on the life of an AYA is required to better support them through any sense of loss of identity, independence and feelings of inadequacy.

## 4 Discussion

Our study illustrates that AYAs with HGG face additional challenges due to the unique sequelae of their diagnosis at the AYA life stage. Unlike many other cancer diagnoses, the symptomology of HGG brings an overwhelming combination of cognitive and physical impairments—such as severe memory loss, difficulty concentrating, seizures, fatigue, and muscle weakness—which severely impacts their ability to navigate key life stage specific goals. These impairments hinder their progression in critical areas like career development, education, and relationships. The resulting disruptions not only impact their independence and identity but also affect their ability to engage in social and life‐stage appropriate activities, leading to isolation and dependency.

Despite the uniqueness of these challenges, AYAs with HGG encounter challenges that are often comparable to those faced by other AYA cancer patients [23]. AYAs cancer patients can benefit from a life‐stage specific care that addresses emotional and psychosocial well‐being, education and career planning, fertility concerns, and financial considerations, cognitive rehabilitation, peer connections, long‐term survivorship planning, and active participation in treatment decisions [24, 25]. The diagnosis and treatment of cancer at this stage in life significantly disrupt critical developmental milestones, such as identity formation, education and career progression, family planning, and relationship building, bringing about psychosocial stressors [26, 27]. Like other AYA cancer patients, those with HGG experience these same challenges [24, 28]. Our interviews illuminated concerns associated with managing cognitive and physical impairments associated with HGG, disruptions in life‐stage specific goals, social isolation, and loss of identity and independence. These are comparable experiences with other AYAs. For example, in a study by Huges et al. similar challenges were identified among AYAs with cancer, emphasizing the need for tailored support to address cognitive changes, physical limitations, and the impact of these factors on social relationships and personal goals [29].

To manage these unique challenges, our participants suggested the development of tailored support programs to address the specific needs of AYAs with HGG. Results indicate that cognitive rehabilitation programs are crucial, as 82% of respondents reported persistent fatigue and 76% experienced cognitive changes that significantly disrupted their education and career goals. Specialized programs focusing on fatigue management and cognitive support could prevent further career and life disruptions. Additionally, support addressing the physical and cognitive impairments that impact social life, such as vision therapy and balance training, is necessary to combat social isolation and relationship difficulties. Psychological support should focus on navigating the loss of identity and independence caused by seizures and mood changes, with resources to manage these effects and provide clarity on long‐term expectations. Integrating these insights into existing literature highlights the need for comprehensive, individualized support systems to improve the quality of life for AYAs with HGG.

The findings of this study offer insights into the specific needs and experiences of AYAs with HGG. While previous research has broadly addressed the challenges faced by AYAs with cancer [30, 31], this study offers new insights into the specific needs and experiences of AYAs with HGG, revealing distinct cognitive, physical, and psychosocial challenges compared to other cancer types like breast cancer or leukemia. AYAs with HGG experience pronounced cognitive impairments, including memory loss and attention deficits, more severe than those associated with other cancers. They also face significant physical issues such as motor deficits and seizures, alongside heightened emotional distress and isolation. In contrast, older adults with HGG contend with age‐related cognitive decline and different coping mechanisms, often having more established support systems [32, 33, 34]. This underscores the need for tailored interventions for AYAs with HGG to address their unique challenges and support requirements effectively.

Support interventions for HGG patients typically include palliative care, neurocognitive rehabilitation, psychosocial support, and caregiver involvement [35]. These interventions aim to manage symptoms like cognitive deficits, emotional distress, and quality‐of‐life deterioration. Studies have shown that while these interventions can improve psychological well‐being and symptom management, their effectiveness in younger populations like AYAs is limited, as they fail to address the unique cognitive and psychosocial challenges AYAs face [36]. Our research offers a comprehensive understanding of the specific support systems required by this population and can contribute to the literature on AYA oncology and emphasize the necessity for specialized care programs tailored to AYAs with HGG.

The study has some notable strengths and limitations. The mixed‐methods approach, which combines quantitative survey data with qualitative interview insights, provides a comprehensive understanding of the participants' experiences. The use of thematic analysis further strengthens the validity of the findings. However, the small sample size of 17 participants may not capture the full spectrum of experiences and needs of all AYAs with HGG. For example, there is a lack of representation from diverse socioeconomic backgrounds and ethnic groups, which could potentially influence the results, as prior research indicates that cultural differences can significantly affect how AYAs perceive and manage their cancer diagnosis and treatment [37]. Patients who agreed to participate in the study may also be unrepresentative of the AYA HGG population as a whole, because they were alive, functional and motivated to participate in this qualitative study. The study's setting at a single tertiary cancer center may limit the transferability of the findings to other contexts [38]. The COVID‐19 pandemic likely affected healthcare delivery and patient experiences [39]. Additionally, the reliance on self‐reported data introduces potential biases, including recall bias and social desirability bias, especially on sensitive topics like satisfaction and resource awareness [40]. The lack of longitudinal data prevents assessing changes over time and long‐term impacts on AYA patients' lives.

Future research with larger, more diverse samples and multi‐center studies would help validate study findings and further elucidate the needs of this population. Next steps should also include developing tailored resources and tools to support AYA patients' unique challenges. Additionally, a planned phase 2 study will create a whiteboard video to highlight these challenges and evaluate its effectiveness in educating about the needs of this vulnerable population.

In conclusion, AYAs with HGG face substantial, unique challenges that significantly impact their quality of life and ability to achieve life‐stage milestones. This study highlights the need for comprehensive, tailored support services to address their cognitive, physical, and psychosocial needs. Implementing specialized programs and resources is essential to enhance the overall well‐being of these young patients and aid them in navigating the complexities of living with HGG. Given the potential for novel targeted and immunotherapies to improve overall survival in HGG, it is crucial to incorporate patient‐reported outcomes, including qualitative interviews, in interventional clinical trials.

## Author Contributions

**Kaviya Devaraja:** conceptualization (lead), data curation (lead), formal analysis (lead), funding acquisition (equal), investigation (lead), methodology (lead), project administration (lead), validation (lead), writing – original draft (lead), writing – review and editing (lead). **Maureen Daniels:** investigation (supporting), project administration (supporting), validation (supporting), writing – review and editing (supporting). **Derek S. Tsang:** conceptualization (supporting), data curation (supporting), investigation (supporting), project administration (supporting), validation (supporting), writing – review and editing (supporting). **Kim Edelstein:** investigation (supporting), project administration (supporting), validation (supporting), writing – review and editing (supporting). **Julie Bennett:** investigation (supporting), validation (supporting), writing – review and editing (supporting). **Cheryl Kanter:** investigation (supporting), validation (supporting), writing – review and editing (supporting). **Warren Mason:** validation (supporting), writing – review and editing (supporting). **Abha A. Gupta:** conceptualization (supporting), data curation (supporting), funding acquisition (equal), investigation (supporting), methodology (supporting), supervision (supporting), validation (supporting), writing – review and editing (supporting). **Jonathan Avery:** conceptualization (supporting), data curation (supporting), formal analysis (supporting), funding acquisition (equal), investigation (supporting), methodology (supporting), supervision (lead), validation (supporting), writing – original draft (supporting), writing – review and editing (supporting).

## Ethics Statement

Ethics approval was obtained from the University Health Network (UHN) research ethics board (CAPCR # 22‐5430).

## Consent

Written, informed consent was obtained from all participants in the study.

## Conflicts of Interest

D.S.T. is a consultant with Need (https://www.getneed.com), unrelated to this work.

## Supporting information


**Table S1.** Online survey: questions.
**Table S2.** Interview guide script: questions and probes.

## Data Availability

The data presented in this study are available on request from the corresponding author.

## References

[1] G. B. Giammalva , D. G. Iacopino , G. Azzarello , et al., “End‐Of‐Life Care in High‐Grade Glioma Patients: The Palliative and Supportive Perspective,” Brain Sciences 8, no. 7 (2018): 1–10, 10.3390/brainsci8070125. PMC6071221 29966347

[2] M. R. Voisin , S. Sasikumar , A. Mansouri , and G. Zadeh , “Incidence and Prevalence of Primary Malignant Brain Tumours in Canada From 1992 to 2017: An Epidemiologic Study,” CMAJ Open 9 (2021): 939–973. 10.9778/cmajo.20200295 PMC8580830 34753786

[3] Brain Tumour Foundation of Canada , “Brain Tumour Types,” Glioblastoma (GB), accessed March 17, 2025, https://www.braintumour.ca/brain_tumour_types/glioblastoma‐gb/.

[4] M. A. Vaz‐Salgado , M. Villamayor , V. Albarran , et al., “Recurrent Glioblastoma: A Review of the Treatment Options,” Cancers 15 (2023): 1–22. 10.3390/cancers15174279 PMC10487236 37686553

[5] F. Yamasaki , “Adolescent and Young Adult Brain Tumors: Current Topics and Review,” International Journal of Clinical Oncology 27 (2022): 457–464. 35064353 10.1007/s10147-021-02084-7 PMC8782686

[6] S. H. M. Janssen , W. T. A. Van der Graaf , D. J. Meer , E. Manten‐Horst , and O. Husson , “Adolescent and Young Adult (AYA) Cancer Survivorship Practices: An Overview,” Cancers 13, no. 19 (2021): 1–23, 10.3390/cancers13194847. PMC8508173 34638332

[7] A. Roux , J. Pallud , R. Saffroy , et al., “High‐Grade Gliomas in Adolescents and Young Adults Highlight Histomolecular Differences From Their Adult and Pediatric Counterparts,” Neuro‐Oncology 22, no. 8 (2020): 1190–1202, 10.1093/neuonc/noaa024. 32025728 PMC7594566

[8] Y. N. Alhalaseh , Z. A. Abdulelah , A. Abu‐Shanab , et al., “Glioblastoma in Adolescents and Young Adults: An Age‐Based Comparative Study From Jordan Over a 17‐Year Period,” Cancer Epidemiology 73 (2021): 1–7, 10.1016/j.canep.2021.101948. 33975256

[9] F. S. M. Schulte , S. H. J. Hou , J. L. Bender , et al., “An Investigation of Social Status Among Adolescents and Young Adults Who Have Been Diagnosed With Cancer in Canada,” Cancers 15, no. 13 (2023): 1–12, 10.3390/cancers15133436. PMC10341042 37444545

[10] A.‐S. Darlington , S. C. Sodergren , O. Husson , et al., “Quality of Life in Adolescents and Young Adults With Cancer,” in Handbook of Quality of Life in Cancer, ed. S. Singer and J. Arraras (Wiley‐Blackwell, 2020), 275–286.

[11] N. Itzep and M. Roth , “Psychosocial Distress due to Interference of Normal Developmental Milestones in AYAs With Cancer,” Children 9, no. 3 (2022): 1–8, 10.3390/children9030309. PMC8947616 35327680

[12] H. D. Druker , K. Zelley , R. D. McGee , et al., “Genetic Counselor Recommendations for Cancer Predisposition Evaluation and Surveillance in the Pediatric Oncology Patient,” Clinical Cancer Research 23 (2017): 91–97. 10.1158/1078-0432.CCR-17-0834 28674117

[13] J. Avery , E. Wong , C. Harris , et al., “The Transformation of Adolescent and Young Adult Oncological and Supportive Care in Canada: A Mixed Methods Study,” Current Oncology 29 (2022): 5126–5138. 35877266 10.3390/curroncol29070406 PMC9321170

[14] U. Hafeez , S. Menon , B. Nguyen , et al., “Young Adults Diagnosed With High Grade Gliomas: Patterns of Care, Outcomes, and Impact on Employment,” Journal of Clinical Neuroscience 68 (2019): 45–50. 31371189 10.1016/j.jocn.2019.07.063

[15] E. Nicklin , L. Pointon , A. Glaser , et al., “Unmet Support Needs in Teenage and Young Adult Childhood Brain Tumour Survivors and Their Caregivers: “It's All the Aftermath, and Then You're Forgotten About”,” Support Care Cancer 29 (2021): 6315–6324. 33861364 10.1007/s00520-021-06193-x PMC8464553

[16] National Cancer Institute , “Adolescent and Young Adult (AYA) Cancer,” U.S. Department of Health and Human Services, accessed August 23, 2024, https://www.cancer.gov/types/aya.

[17] S. Rahimi and M. Khatooni , “Saturation in Qualitative Research: An Evolutionary Concept Analysis,” International Journal of Nursing Studies Advances 6 (2024): 1–11, 10.1016/j.ijnsa.2024.100174. PMC11080421 38746797

[18] A. W. Smith , T. Keegan , A. Hamilton , et al., “Understanding Care and Outcomes in Adolescents and Young Adults With Cancer: A Review of the AYA HOPE Study,” Pediatric Blood & Cancer 66 (2019): 1–7. 10.1002/pbc.27486 PMC7239374 30294882

[19] H. S. Markwardt , S. E. Taghavi , A. P. Williams , et al., “The AYA Care Plan: Initial Evaluation of a Web‐Based Psychosocial Intervention,” JCO Clinical Cancer Informatics 6 (2022): 1–6. 10.1200/CCI.22.00086 36306498

[20] S. Jamshed , “Qualitative Research Method‐Interviewing and Observation,” Journal of Basic and Clinical Pharmacy 5, no. 4 (2014): 87–88, 10.4103/0976-0105.141942. 25316987 PMC4194943

[21] B. Kim , K. White , and P. Patterson , “Understanding the Experiences of Adolescents and Young Adults With Cancer: A Meta‐Synthesis,” European Journal of Oncology Nursing 24 (2016): 39–53. 27697276 10.1016/j.ejon.2016.06.002

[22] V. Braun and V. Clarke , “Using Thematic Analysis in Psychology,” Qualitative Research in Psychology 3 (2006): 77–101.

[23] C. Link , S. Qi , S. Thompson , A. Delure , S. McKillop , and L. Watson , “Understanding the Symptoms and Concerns of Adolescents and Young Adults With Cancer in Alberta: A Comparative Cohort Study Using Patient‐Reported Outcomes,” Journal of Adolescent and Young Adult Oncology 12, no. 2 (2023): 199–206, 10.1089/jayao.2021.0213. 35749720 PMC10124169

[24] R. S. Fox , G. E. Armstrong , J. S. Gaumond , et al., “Social Isolation and Social Connectedness Among Young Adult Cancer Survivors: A Systematic Review,” Cancer 129 (2023): 2946–2965. 37489837 10.1002/cncr.34934 PMC10584376

[25] E. Lidington , A. S. Darlington , A. Din , et al., “Describing Unmet Supportive Care Needs Among Young Adults With Cancer (25–39 Years) and the Relationship With Health‐Related Quality of Life, Psychological Distress, and Illness Cognitions,” Journal of Clinical Medicine 10 (2021): 1–14. 10.3390/jcm10194449 PMC8509768 34640467

[26] J. A. Hydeman , O. C. Uwazurike , E. I. Adeyemi , and L. K. Beaupin , “Survivorship Needs of Adolescent and Young Adult Cancer Survivors: A Concept Mapping Analysis,” Journal of Cancer Survivorship: Research and Practice 12 (2019): 32–42. 10.1007/s11764-018-0725-5 PMC6699632 30604138

[27] S. Brand , J. Wolfe , and C. Samsel , “The Impact of Cancer and Its Treatment on the Growth and Development of the Pediatric Patient,” Current Pediatric Reviews 13 (2017): 24–33. 27848890 10.2174/1573396313666161116094916 PMC5503788

[28] B. A. Sisk , K. Fasciano , S. D. Block , and J. W. Mack , “Impact of Cancer on School, Work, and Financial Independence Among Adolescents and Young Adults,” Cancer 126 (2020): 4400–4406. 32658324 10.1002/cncr.33081 PMC7719071

[29] L. Hughes , R. M. Taylor , A. E. Beckett , et al., “The Emotional Impact of a Cancer Diagnosis: A Qualitative Study of Adolescent and Young Adult Experience,” Cancers 16, no. 7 (2024): 1–21, 10.3390/cancers16071332. PMC11010824 38611010

[30] S. E. J. Kaal , E. K. Lidington , J. B. Prins , et al., “Health‐Related Quality of Life Issues in Adolescents and Young Adults With Cancer: Discrepancies With the Perceptions of Health Care Professionals,” Journal of Clinical Medicine 10, no. 9 (2021): 1–15, 10.3390/jcm10091833. PMC8122798 33922382

[31] S. Hovsepyan , J. Hoveyan , L. Sargsyan , et al., “The Unique Challenges of AYA Cancer Care in Resource‐Limited Settings,” Frontiers in Adolescent Medicine 1 (2023): 1–6, 10.3389/fradm.2023.1279778.

[32] S. K. Janardan and D. S. Wechsler , “Caught in the In‐Between: Challenges in Treating Adolescents and Young Adults With Cancer,” JCO Oncology Practice 17 (2021): 299–301. 33914610 10.1200/OP.21.00178 PMC8258002

[33] M. A. Kirkman , B. H. M. Hunn , M. S. C. Thomas , and A. K. Tolmie , “Influences on Cognitive Outcomes in Adult Patients With Gliomas: A Systematic Review,” Frontiers in Oncology 12 (2022): 1–23, 10.3389/fonc.2022.943600. PMC9407441 36033458

[34] R. A. Morshed , J. S. Young , A. A. Kroliczek , M. S. Berger , D. Brang , and S. L. Hervey‐Jumper , “A Neurosurgeon's Guide to Cognitive Dysfunction in Adult Glioma,” Neurosurgery 89 (2021): 1–10. 33289504 10.1093/neuros/nyaa400 PMC8203420

[35] R. C. Crooms , N. E. Goldstein , E. L. Diamond , and B. G. Vickrey , “Palliative Care in High‐Grade Glioma: A Review,” Brain Sciences 10, no. 10 (2020): 1–26, 10.3390/brainsci10100723. PMC7599762 33066030

[36] E. M. Byrne , M. Pascoe , D. Cooper , T. S. Armstrong , and M. R. Gilbert , “Challenges and Limitations of Clinical Trials in the Adolescent and Young Adult CNS Cancer Population: A Systematic Review,” Neuro‐Oncology Advances 6, no. 1 (2024): 1–16, 10.1093/noajnl/vdad159. PMC10798804 38250563

[37] S. H. J. Hou , A. Petrella , J. Tulk , et al., “An Evaluation of Racial and Ethnic Representation in Research Conducted With Young Adults Diagnosed With Cancer: Challenges and Considerations for Building More Equitable and Inclusive Research Practices,” Current Oncology 31, no. 4 (2024): 2244–2259, 10.3390/curroncol31040166. 38668069 PMC11048902

[38] R. E. Stalmeijer , M. E. L. Brown , and B. C. O'Brien , “How to Discuss Transferability of Qualitative Research in Health Professions Education,” Clinical Teacher 1, no. 1 (2024): 1–7. 10.1111/tct.13762 38497107

[39] M. Barankova , K. Greskovicova , B. Strnadelova , K. Krizova , and J. Halamova , “Let us Take It Into Our Own Hands: Patient Experience During the COVID‐19 Pandemic,” International Journal of Environmental Research and Public Health 19, no. 21 (2022): 1–15, 10.3390/ijerph192114150. PMC9655667 36361026

[40] N. Bergen and R. Labonte , ““Everything Is Perfect, and we Have no Problems”: Detecting and Limiting Social Desirability Bias in Qualitative Research,” Qualitative Health Research 5 (2019): 783–792. 10.1177/1049732319889354 31830860

