# Supplementary material for: Navigating Life With High‐Grade Glioma: Experiences and Needs of Adolescents and Young Adults
Source: Cancer Med. 2025 Apr 8;14(7):e70867. doi: 10.1002/cam4.70867 (PMC11976453; doi:10.1002/cam4.70867)
Supplement: Supplementary file 1 — Table S1. Online survey: questions. Table S2. Interview guide script: questions and probes. [file CAM4-14-e70867-s001.docx]

Supplementary Files

Supplemental Table 1: Online survey: questions

| Part 1: Demographics |  | | |
| --- | --- | --- | --- |
| Question | Response Choices | | |
| 1. Date of birth | Month/year  I prefer not to answer | | |
| 1. Where were you born? | Canada  Other, please specify  I prefer not to answer | | |
| 1. What is your racial or ethnic background? (select all that apply) | Arab First Nations Latin American  Black Inuit Metis  Chinese Japanese South Asian  Filipino Korean Southeast Asian  White West Asian Other, please specify  I prefer not to answer | | |
| 1. What is your gender? | Man Non-binary  Woman Two-spirit  Transgender woman Other, please specify  Transgender man I prefer not to answer | | |
| 1. Please select the option that best describes where you live | Rural (population of less than 50,000)  Small town (population from 50,000 to 250,000)  Large city (population from 250,000 to 1 million)  Metropolitan Centre (population of 1 million or more)  Other, please specify  I prefer not to answer | | |
| 1. What is your highest degree or level of school completed | No schooling Graduate/professional degree  Highschool diploma Other, please specify  University/college degree I prefer not to answer | | |
| 1. When were you first diagnosed with cancer? | Less than a year ago More than 6 years ago  1-3 years ago Other, please specify  4-6 years ago I prefer not to answer | | |
| 1. Which of the following best describes where you are with your care/treatments? | You have yet to start cancer treatment  You are currently in active treatment  You have completed treatment and in long-term follow-up  You have been discharged from your cancer centre  You are unsure  Other, please specify  I prefer not to answer | | |
| 1. Please select all the treatments that you have received | Chemotherapy Clinical trials  Radiation Other, please specify  Surgery I prefer not to answer | | |
| 1. What other health conditions do you have? | Diabetes High cholesterol  Hypertension Heart disease  Obesity HIV/AIDs  Another cancer diagnosis Mental illness  Eating disorders Drug & Alcohol abuse  Bronchitis Pneumonia  Thyroid STI/STD  Other, please specify I prefer not to say  Chronic obstructive pulmonary disease (COPD) | | |
| 1. How long have you been a patient at Princess Margaret Cancer Centre or under the care of Princess Margaret? | 0-6 months Over 6 years  6-12 months Other, please specify  1-3 years I prefer not to answer  4-6 years | | |
| 1. What is your employment status | Full-time Other, please specify  Part-time I prefer not to answer  On disability | | |
| 1. What is your yearly household income before taxes? | Less than $30,000 $215,000-$299,999  $30,000-$60,999 Above $300,000  $61,000-$90,000 Other, please specify  $90,000-$150,000 I prefer not to answer  $151,000-$214,999 | | |
| Part 2: AYA Care at Princess Margaret Cancer Centre |  | | |
| 1. What changes or concerns do you experience as a high-grade glioma patient? Please rank the following from 1-6. | 1. Headaches 2. Loss of Appetite 3. Problems with Speech 4. Cognitive changes 5. Changes in mood, behaviour & personality 6. Muscle weakness 7. Visual deficits 8. Seizures 9. Nausea & Vomiting 10. Loss of balance & difficulty walking 11. Body image 12. Sexuality/intimacy 13. Fertility/fertility preservation 14. Urinary incontinence 15. Fatigue | | Frequency of symptom experience   - Not at all - Rarely - Sometimes/ Occasionally - Frequently/ Often - Usually/ Almost Always - Always - I prefer not to answer |
| 1. Are there other symptoms that you experienced with your high-grade glioma? | No  Yes, please specify  I prefer not to answer | | |
| 1. For each statement below, please indicate which option best fits your experience | 1. The education about my diagnosis I received from my health care team, was it: 2. The information I received about symptoms of being diagnosed for high-grade glioma from my healthcare team, was it: 3. The education I received from my healthcare team about available treatment options for high-grade glioma, was it: 4. The education I received from my healthcare team on the resources and support available to me, was it: | | - Not enough - Just enough - Too much - I have yet to receive any detail - I prefer not to answer |
| 1. Please choose the option that best represents your experience: Living with high-grade glioma as a young adult is extremely challenging | Strong agree Neither agree not disagree  Agree Other, please specify  Disagree I prefer not to answer  Strong disagree | | |
| 1. Are you satisfied with the support you received at the Princess Margaret Cancer Centre during you: | 1. Diagnosis 2. Treatment 3. Post-treatment | - Yes - No - I am not sure - I prefer not to answer | |
| 1. Do you feel there is anything missing in the care you are receiving as a young adult high-grade glioma patient receiving treatment and/or post treatment care at the Princess Margaret Cancer Centre? | Yes  No  Other, please specify  I prefer not to answer | | |
| 1. We would like to understand what type of support you have accessed and/or are interested in accessing. Please select the statement that best applies to you: | 1. Princess Margaret Cancer Centre Patient Information Line (info line) 2. AYA Meet-Ups 3. AYA newsletter 4. AYA social media (Twitter, Instagram and Facebook) 5. Young Adult Cancer Canada programs (on-line community, retreats) 6. Princess Margaret specific social media 7. Princess Margaret Patient & Family Library 8. Canadian Cancer Society Community Services 9. Princess Margaret Patient Education Materials 10. Psychosocial oncology service/ program at Princess Margaret 11. Wellspring 12. Gilda’s club 13. Brain tumour foundation of Canada 14. Other (not on list), please specify: | | - I haven’t heard of the program - I’ve heard of the program an am not interested in attending - I’ve heard of the program and am interested (but have yet to attend) - I’ve used/attended the program - I’ve used/attended the program but am no longer interested in attending |

Supplemental Table 2: Interview guide script: questions and probes.

| Part 1 – AYA HGG Experience  Question   - What is it like to live with high-grade glioma as a young adult?   Probes:   1. Do you face any challenges? If so, what are the challenges you face? |
| --- |
| Part 2 – AYA HGG Education  Question   - What is your level of understanding of your high-grade glioma diagnosis?   Probes:   - 1. Is there anything you wish you knew more about? Can you expand?   2. What do you know regarding its impact, symptoms, diagnosis, and treatments available?   3. What do you know about the number of new cases of disease, number of people already with illness, death rate or sickness rate of high-grade glioma among young adults in Canada?      1. Worldwide?      2. The differences between patients based on age. Differences between young adults and older adults         1. Gender         2. Ethnicity         3. Income, education, and occupation   4. What are your opinions on education and awareness of high-grade glioma among the young adult community?      1. What changes would you like to see in young adult high-grade glioma education, awareness, and advocacy? |
| Part 3 – AYA HGG Cancer Care  Question   - What are your personal experiences as a patient at this cancer centre ?   Probes:   - 1. What are your thoughts and opinions of young adult high-grade glioma research at the cancer centre?   2. What do you think is done well at the cancer centre in terms of diagnosis, treatment, resources made available, and research conducted on young adult high-grade glioma patients?      1. What gaps exist?      2. What changes would you like to see?   3. What are your thoughts and opinions about the amount of research and resources directed towards this community?   4. Are there any gaps that exist? Can you speak about any gaps that exist?   5. Are there any changes you would like to see? What changes would you like to see? |
| Part 4 – AYA HGG Specific Resources and Support  Question   - What resources, and/or support services are you accessing the cancer centre?   Probes:   - 1. What are your opinions on the current services made available?      1. Are there any gaps that exist? If yes, what are these gaps?      2. Are there any changes you would like to see? If yes, what changes would you like to see? Why?   2. Are there any types of services/support would you like to see? Can you elaborate on what these services might look like?   3. If any, what tools do you have access to at the cancer centre?      1. Do these tools educate you on high-grade glioma diagnosis, treatment options, statistics, research and resources available to young adult patients?      2. Are there tools you would you like access to in order to improve your overall health care experience, course of disease, quality of life?         1. If yes, what would these tools include?         2. Would you like the tool to allow you to connect to other patients? Why or why not? |
| Final Comments   - Is there anything else that you would like to add? Is there any important issue that we have not talked about and that you would like to share with me? |
